# EpiScanGIS: an online geographic surveillance system for meningococcal disease

**DOI:** 10.1186/1476-072X-7-33

**Published:** 2008-07-01

**Authors:** Markus Reinhardt, Johannes Elias, Jürgen Albert, Matthias Frosch, Dag Harmsen, Ulrich Vogel

**Affiliations:** 1 Computer Science II, University of Würzburg, Germany; 2 Institute for Hygiene and Microbiology, University of Würzburg, Germany; 3 Department of Periodontology, University of Münster, Germany; 4 Ridom GmbH, Würzburg, Germany

## Abstract

**Background:**

Surveillance of infectious diseases increasingly relies on Geographic Information Systems (GIS). The integration of pathogen fine typing data in dynamic systems and visualization of spatio-temporal clusters are a technical challenge for system development.

**Results:**

An online geographic information system (EpiScanGIS) based on open source components has been launched in Germany in May 2006 for real time provision of meningococcal typing data in conjunction with demographic information (age, incidence, population density). Spatio-temporal clusters of disease detected by computer assisted cluster analysis (SaTScan™) are visualized on maps. EpiScanGIS enables dynamic generation of animated maps. The system is based on open source components; its architecture is open for other infectious agents and geographic regions. EpiScanGIS is available at , and currently has 80 registered users, mostly from the public health service in Germany. At present more than 2,900 cases of invasive meningococcal disease are stored in the database (data as of June 3, 2008).

**Conclusion:**

EpiScanGIS exemplifies GIS applications and early-warning systems in laboratory surveillance of infectious diseases.

## Background

The incidences of transmissible infectious diseases display considerable fluctuation in time and space. Forecasts are hampered by multiple influencing factors, such as virulence of causative agents and their genetic variants, social networks and travel, herd immunity, and climate changes.

Members of the epidemic intelligence community increasingly rely on geographic information systems (GIS) to assess outbreaks in real time and to counsel decision makers regarding the implementation of control measures. GIS visualize complex spatio-temporal events and thus help to analyze data on geographic maps consisting of several layers of information. Data can be shared over local computer networks or via the internet. In contrast to traditional maps, GIS are updateable, and help to appropriately target intervention and prevention programmes, especially in less developed countries [1,2].

The WHO launched a public health mapping GIS programme in 1993 [3]. In addition, the revised WHO International Health Regulations (IHR 2005, Annex 1) set out core capacities, e.g. use of early warning systems and most efficient information technologies, to be implemented by member states for improved detection and surveillance of health threats including meningococcal disease. National GIS programmes for surveillance of communicable diseases can be accessed via the internet, such as the Swedish SmiNet, which provides infection epidemiological data in various formats including a GIS [4]. The German SurvStat@RKI displays data on notifiable diseases in Germany not only as tables, but also chloropleth maps can be generated [5]. GIS have also been established to monitor infectious disease spread and target intervention strategies in more refined geographic units such as military installations [6], hospitals [7], or cities [6].

A variety of specialized applications for scientific and public health purposes have been introduced. Animated maps showing geographic movements of influenza waves throughout a country inform the public about current trends and foster efforts to promote vaccination [8]. A web GIS based on open source components has been launched for Canadian West Nile virus surveillance, which makes information on dead birds readily available to the private and public sector [9]. Environmental information can be integrated into GIS to study environmental triggers and other risk factors for infectious diseases [10-17]. For this purpose, satellite imagery and remote sensing have become increasingly important [18,19]. All these projects demonstrate the capacity of GIS to integrate, evaluate, and visualize tremendous amounts of multi-purpose data. GIS projects are also employing historical datasets to unravel the transmission patterns of infectious diseases [20]. Fine typing of pathogens, a method to define groups or even clones within a given species, can provide an additional layer of information, as exemplified for avian malaria [21]. Scan statistics has been used to complement GIS with regard to spatio-temporal cluster detection [22-24].

Meningococcal disease is caused by the Gram negative bacterium *Neisseria meningitidis*, which poses a substantial threat to childhood health throughout the world [25]. Case fatality rate reaches 10% even in countries with excellent health care, there is a high rate of sequelae, and transmission patterns and outbreak sizes are rather unpredictable. Unexpected increases of reported cases of disease have been observed in several countries, giving rise to tremendous efforts for vaccine development or distribution [26]. A variety of clonal lineages of meningococci occur world-wide with varying distribution [27]. Whereas these lineages are defined by multilocus sequence typing or formerly multilocus enzyme electrophoresis [28], fine typing of meningococci for outbreak detection is achieved by DNA-sequence typing of variable regions of genes encoding immuno-dominant antigens [29,30]. We recently used fine typing data to identify clusters of meningococcal disease using automated scan statistics [31,32]. The combination of reliable DNA-sequence typing with scan statistics provides an unbiased approach for cluster detection and early warning of public health agencies.

Here we report the development and implementation of a meningococcal disease GIS, which visualizes the dynamics of the spread of meningococcal disease including results of scan statistics.

## Results

An epidemiological GIS was developed which provides timely access to laboratory surveillance data on meningococcal disease in Germany, and which supplements statutory meningococcal disease surveillance by the Federal Health Authorities. All technical components of EpiScanGIS are described in detail in the Materials and Methods section.

**Figure 1:**
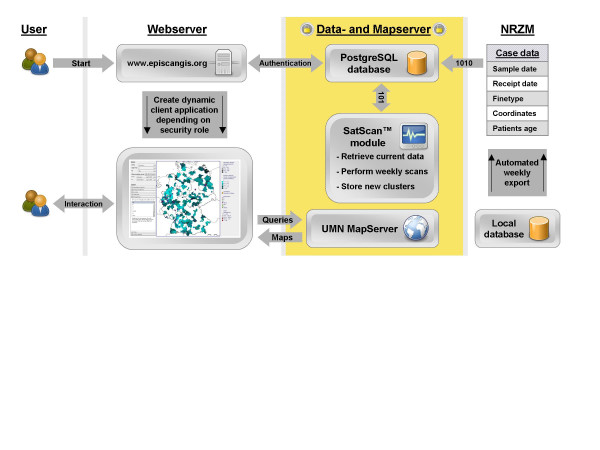
**Structure of EpiScanGIS, an online geographical information system for meningococcal disease surveillance in Germany. **EpiScanGIS generates a Flash-based Rich Internet Application, which is delivered to the user upon connection. The graphical user interface (GUI) is initialized dynamically depending on the user's security role. The GUI forwards all interactions to the server-side controller and receives and displays the appropriate map and information. Technical details of the application are described in the Materials and Methods section.

EpiScanGIS comprises a data- and a mapserver and a publicly available web server. Data transmission, database content, and features of visualization were designed to offer the highest possible level of data protection. Only registered users may generate enlarged maps of single Federal States with enhanced resolution, and are allowed to access disease cluster information. (Currently, 80 users mainly from the public health sector have been registered). The architecture of EpiScanGIS is depicted in Fig. 1.

The German reference laboratory for meningococci (NRZM) provides comprehensive laboratory surveillance for meningococcal disease in Germany [33]. Data of the NRZM stored in a local database are synchronized with EpiScanGIS on a weekly basis. Date of sampling, date of receipt, geographic coordinates as deduced from the postal code, age of patient, and fine type of the meningococcus are automatically transferred. An interactive internet application allows users to generate maps dynamically for answering queries adjusted to an arbitrary period, age group, county, serogroup or fine type. The system is able to show different map layers, which display population density, yearly incidence, cases grouped by serogroup or fine type, and the results of cluster analysis, in any possible combination. Additional information on specific coordinates is extractable by pointing on the respective map position.

**Figure 2:**
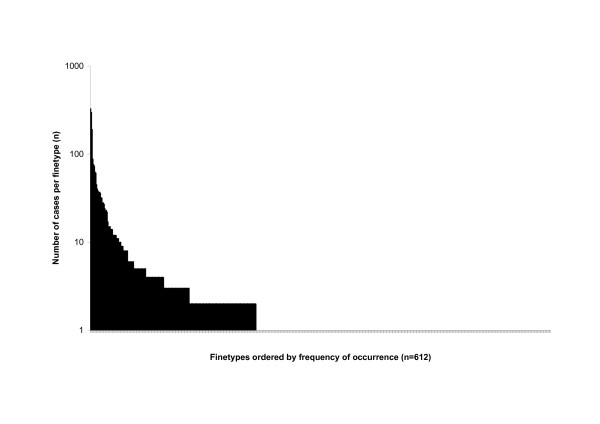
**Rank abundance curve demonstrating the frequency of cases (y-axis) belonging to one of 612 distinct fine types, which are stored in the EpiScanGIS database.** The most frequent fine type is B:P1.7-2,4:F1-5 (n = 328 in April 2008); 391 of 612 fine types occurred only once (December 2001 through April 2008).

**Figure 3:**
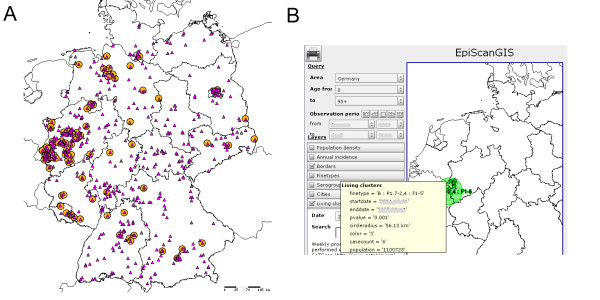
**Examples for the use of EpiScanGIS.** (A) For the years 2004 and 2005, a query was made for a very frequent meningococcal serogroup B fine type (B:P1.7-2,4:F1-5), whose distribution (circles) is compared with all serogroup B cases (triangles) in the database. Note that serogroup B cases due to this fine type are mostly found in the Western part of Germany. (B) Section of the EpiScanGIS screen display: a spatio-temporal cluster is depicted by a circle surrounding the location of cases due to a certain fine type (B:P1.7-2,4:F1-5), which are in close spatio-temporal proximity. A description field provides additional information on the cluster. Temporal information has been obscured for the purpose of publication.

As a tool for laboratory surveillance of infectious diseases, EpiScanGIS incorporates a defined set of bacterial fine typing data. Consequently, case information is only included, if a complete fine typing dataset is available. DNA-sequence based typing information was chosen, because of its accuracy and reproducibility [29]. Currently, more than 2,900 cases have been compiled in the database, with a total of 612 fine types (as defined by a unique combination of capsular serogroup, outer membrane protein PorA type and outer membrane protein FetA type (Figure 2). Figure 3A depicts a typical query for a specific fine type (B:P1.7-2,4:F1-5 with B, serogroup; P1., PorA type; F, FetA type) in comparison to any other serogroup B strain.

In EpiScanGIS, pathogen specific information is embedded in generic attributes. The database does not store a fine type character as a fixed column with each case record, but connects the latter with a generic attribute. The case record itself only contains a minimal demographic dataset. Specific attributes are generated on the fly upon data import. Thus, EpiScanGIS is an open platform, which may be adopted for any other infectious agent.

EpiScanGIS is connected to an automated cluster detection system. We chose SaTScan for this purpose [34-36], which we validated recently [31]. SaTScan is employed on weekly basis; possible spatio-temporal clusters of meningococcal cases caused by identical fine types are visualized on the maps. EpiScanGIS extracts relevant information such as number of cases, fine type, county, p-value, and diameter of the circle, in which the abnormal accumulation of cases occurred. Figure 3B demonstrates the visualization of a cluster. As described recently, cluster analysis is initiated at the centroids of the 429 rural and urban districts, Germany's administrative units.

EpiScanGIS enables real-time online animation that can be used to show time-lapse videos of arbitrary maps with flexible time-period adjustment. Such animations are helpful to visualize spatial distribution of cases caused by a given fine type over time.

## Discussion

Meningococcal disease is monitored by national and international surveillance systems in many parts of the world. Surveillance is necessary to assess the disease burden and to appropriately allocate resources for preventive measures, such as vaccine development. Fine typing procedures are important for monitoring of clonal spread and distribution, outbreak monitoring, and for the assessment of vaccine coverage against this highly variable organism [37].

A variety of characteristics make meningococcal disease an ideal candidate for GIS applications. Firstly, meningococcal disease is transmitted mostly within the community via close contacts between humans. Nosocomial transmission is an exceptional observation [32], in contrast to e.g. Methicillin-resistant *Staphylococcus aureus *(MRSA), for which geographic mapping might be much more difficult, since a considerable proportion of cases are health care associated. The social networks, within which meningococcal disease is transmitted, are at least partially understood. Household transmission is of major importance, given the fact that risk of secondary infection is highest in this setting [38,39]. Institutional transmission is much less common, yet can occur in schools [40], and military camps [41]. Travel associated meningococcal disease has been reported [42,43], and certainly, detection of travel associated outbreaks is demanding because the geographic location of pathogen acquisition frequently remains obscure. But again, travel associated disease is rare in comparison to the large number of cases acquired within other social networks. Secondly, invasive meningococcal disease is rare. Because of the severity of disease, the high rate of hospitalization, and the relative ease of disease recognition, underreporting is comparatively low, and laboratory confirmation is sought for in the majority of cases. In contrast, other infections such as pneumococcal disease or MRSA-related infections occur at a high frequency with a plethora of disease entities, and in many countries there is no mandatory notification of cases. Thirdly, a consensus on molecular typing of meningococcal disease has recently been reached in Europe [29]. Thus, an extension of EpiScanGIS to other countries with a functioning laboratory surveillance of meningococcal disease put in place will be possible without changing typing attributes.

The provided GIS solution would not have been possible without the use of open source software. Using as much existing tools as possible not only reduces the time of development, but also increases software stability and security. Advanced open source projects are characterized by having many developers and frequent releases, whereby errors are detected and fixed very fast. Furthermore, components can be used free of charge. Given the fact that the infectious disease burden in developing countries with low economic resources is especially high, open source based systems for epidemiological surveillance may be introduced to assist in prompt identification of high risk regions and outbreaks. Provided that samples are linked to geocoding data, EpiScanGIS could easily be implemented in the African meningitis belt for surveillance projects and outbreak management. Not surprisingly, the use of open source components has found its way into health geographics [44].

The majority of samples submitted to the reference laboratory are provided by microbiological laboratories, which send bacterial strains on a voluntary basis. The results of the reference laboratory are reported back, which is essential to involve the peripheral laboratories. However, apart from the capsular serogroup, which might support individual patient management, the fine typing data themselves are of no specific interest to the sending institution. EpiScanGIS for the sender of specimens now offers the opportunity to query the database and extract data on the geographical distribution of the reported fine type, helping the sender to better understand the mission and benefits of laboratory surveillance. Thus, EpiScanGIS will help to stabilize the relation between sending laboratories and the reference laboratory, and thereby it will maintain a high coverage of cases by the laboratory surveillance system. Another issue is of similar importance in this context: weekly written reports of the reference laboratory to the public health offices on newly detected clusters of meningococcal disease need to be supplemented with geographical maps depicting the location of the clusters and data on the general distribution of the fine type under investigation. All of this can be accomplished using EpiScanGIS, and therefore the system has become an essential part of the reporting workflow.

EpiScanGIS still has limitations that will be subject to future modifications. EpiScanGIS only accepts fully typed cases of meningococcal disease. It would be desirable to further include cases in which e.g. for various reasons only the serogroup could be determined by the laboratory, but not PorA- and FetA antigen types. Furthermore, the Robert Koch-Institute in collaboration with the NRZM currently develops algorithms to match the datasets of the RKI (statutory notification system) and of the NRZM, which are not fully overlapping. Inclusion of validated data from the statutory notification system not present in the NRZM dataset would be highly desirable to fully represent the epidemiology of meningococcal disease. At present, in EpiScanGIS incidence calculations are not possible for specific fine types, but only for capsular serogroups. A further adaptation of the system in this sense would facilitate comparisons between fine types of interest. Finally, the space-time scan statistics used and visualized by EpiScanGIS could be improved in future versions, i.e. by initiating the spatial scanning window at a more refined position on the map rather than at the centroid of the administrative unit, which was decided for to reduce the computing time, despite of the fact that the coordinates of the cases are available through the 5-digit postal code. Furthermore, changing to a flexible shaped scan statistic, as described recently [45,46], might be beneficial.

In summary, automated computer assisted cluster detection of meningococcal disease has recently been introduced into the German reference laboratory for meningococci [31]. The system is now used prospectively to identify living clusters of disease caused by a single fine type. Data are reported to the local health offices, the Federal State offices, and the Robert Koch-Institute. To our experience cluster reporting fosters the dialogue between neighbouring public health offices, and initiates exchange of information between various levels of public health administration.

## Conclusion

EpiScanGIS has become a valuable tool for real time laboratory surveillance of meningococcal disease in Germany with the potential to serve as an early warning system. It serves as a model for future GIS applications in infectious disease control. EpiScanGIS has the potential to be extended to an international level and used for other infectious diseases. Depending on the application, further levels of information might be included, such as climate data, socio-demographic data, and risk factors attributable.

## Methods

### System architecture

EpiScanGIS was developed using Java 2 Standard Edition [47]. A PostgreSQL server [48] provides the relational database backend. Apache and Jakarta Tomcat web server [49] deliver the website. MapServer [50] and PostGIS [51] are responsible for the GIS functionality. The web module is built using the Struts application framework [52]. Our Flash-based Rich Internet Application (RIA) employs the OpenLaszlo platform.

### Data acquisition and processing

The German reference laboratory for meningococci (NRZM) provides comprehensive laboratory surveillance for meningococcal disease in Germany [33]. Since 2002, the NRZM maintains a protected database for local access, which stores laboratory findings as well as personal information of each patient. A PostgreSQL database structure was developed that is supplied with the dates of sampling and specimen receipt, the meningococcal fine type, geographical coordinates of the patients' domiciles, and the patients' age at onset of infection. New patients are added to the database on a weekly basis. The selected NRZM data are exported to a simple text format, and transferred to EpiScanGIS using a secure channel in the local area network of the NRZM. The data are automatically synchronized with the PostgreSQL database and are immediately available online.

### Geographical information system

The PostgreSQL extension PostGIS acts as GIS backend, i.e. it provides functions to store and query geographic data. UMN MapServer performs interactive map visualization. The user requests digital maps using a web application, which allows navigating the map and querying available information layers. A new Flash-based application for this task was programmed using the OpenLaszlo platform for web applications. An additional interface combines the web frontend with PostgreSQL, PostGIS and MapServer. It encapsulates the GIS backend and builds the skeletal structure for the website. This interface was built using the Struts application framework, thus gaining the advantages of an extensible Model 2 architectural pattern, which effectively separates presentation from content and the server-side controller. The system's architecture is depicted in Fig. 1.

### Automated cluster detection

The free software SaTScan which was developed by Martin Kulldorff together with Information Management Services Inc. [34-36,53] has been used at the NRZM for detecting spatio-temporal clusters of meningococcal disease in retrospect [31]. In the present paper, a server-side solution implementing this method is described. After weekly transmission of the NRZM dataset, the server processes the computationally expensive cluster analysis on each distinct fine type. The date of submission to NRZM is used if the date of sampling could not be determined. Each fine type is analyzed separately. The prospective analysis is configured to detect clusters of a maximal duration of 60 days containing up to seven percent of the population at risk. In comparison to the recent retrospective analysis over a period of 42 months, the cluster length was extended from 30 to 60 days to increase sensitivity. The centroids of the more than 400 German counties are employed to initiate spatio-temporal scanning. EpiScanGIS parses SaTScan's output files and stores each detected cluster in the database, whereby it can immediately be displayed on maps.

### Data security and confidentiality

The separation of PostgreSQL and MapServer from the web server enforces data security, just as the restriction of the stored data values to date of infection and receipt of specimen, meningococcal fine type, coordinates of the patients' domicile and age of the patient in years. Furthermore, EpiScanGIS protects sensible data from unauthorised users by means of three user roles with respective access constraints. Public access solely allows generating maps at the national level without any information on detected clusters. A member of the public health community may, after authentication, additionally access maps at federal state level including the most recent cluster report. Few members of the NRZM have the highest privileges, including administrative and experimental features. The level of information displayed resembles that of the national statutory data release by the Robert Koch-Institute (SurvStat@RKI) [5]. A firewall and restrictive security policies enforced by security enhanced Linux [54] additionally protect the web server.

## Competing interests

The authors declare that they have no competing interests.

## Authors' contributions

MR development of the GIS as a computer scientist, drafting of the manuscript, JE acquisition of data, development of concepts of integration of scan statistics, revision of the manuscript, JA conception and design of the GIS, revision of the manuscript, MF conception and design of the GIS, revision of the manuscript, DH conception and design of the GIS, revision of the manuscript, UV conception and design of the GIS, drafting of the manuscript.
